# Learning to work and working to learn: a phenomenographic perspective on the transition from student to doctor

**DOI:** 10.1007/s10459-025-10424-9

**Published:** 2025-03-13

**Authors:** Yvonne Carlsson, Filip Olow, Stefan Bergman, Anna Nilsdotter, Matilda Liljedahl

**Affiliations:** 1 School of Public Health and Community Medicine, Institute of Medicine, Sahlgrenska Academy, University of Gothenburg, Gothenburg, Sweden; 2 Department of Accident & Emergency Medicine, NU Hospital Group, Trollhättan, Sweden; 3 Spenshult Research and Development Centre, Halmstad, Sweden; 4 Department of Orthopaedics, Sahlgrenska University Hospital, Gothenburg, Sweden; 5 Department of Oncology, Institute of Clinical Sciences, Sahlgrenska Academy, University of Gothenburg, Gothenburg, Sweden; 6 Department of Oncology, Sahlgrenska University Hospital, Gothenburg, Sweden

**Keywords:** Programme directors, Transition, Medical internship, Phenomenoghraphy, Postgraduate education

## Abstract

The transition from being a medical student to working as a doctor is a pivotal phase, often marked by challenges in balancing learning with the demands of clinical practice. Despite extensive research on the struggles faced by junior doctors, there remains a gap in understanding how other key stakeholders perceive this transition and how it can be viewed as more than just a struggle. In this phenomenographic study, we used the Swedish medical internship as a proxy for the transition and explored internship programme directors’ (PDs) perceptions of the medical internship from a developmental point of view. A phenomenographic approach was chosen to capture the variation in how PDs conceptualise the meaning of the internship, offering a more nuanced understanding of the transition and its implications for educational practice. Interviews with twelve PDs gave rise to three perceptions: the internship as an education, as working as a doctor, and as a space for learning through work. These views highlighted the transition not merely as a preparatory phase but as a dynamic process in which learning and clinical work were intertwined. Our findings suggest that instead of focusing solely on better preparing students for work, empowering junior doctors to learn *through* work—supported by structured guidance—can turn this challenging period into an opportunity for professional and personal growth. This study offers a novel contribution by shedding light on the role of PDs in shaping the transition to clinical work and emphasising the need to view it as a learning-centred, reflective experience.

## Introduction

The transition from medical school to clinical practice is often characterised as a stressful and even threatening phase for junior doctors (Brennan et al., 2010; Prentice et al., 2020; Sitobata & Mohammadnezhad, 2022). Common narratives focus on the difficulties faced by junior doctors during this period, with much of the literature addressing strategies to ‘better prepare’ students for practice and ease the transition (Lyss-Lerman et al., 2009; Pellegrini et al., 2020; Roberts, 2023; Suliman et al., 2023). However, the constantly changing demands of healthcare make it difficult to fully prepare students for practice (Aggarwal et al., 2023; Yardley et al., 2018). By focusing too much on the challenges, we risk overlooking the positive and developmental dimensions of this phase (Atherley et al., 2019). Moreover, framing the transition solely as a struggle might overshadow its transformative potential, essential for professional growth and development as a junior doctor (O’Brien, 2018).

This has led to an alternative approach to researching the transition, viewing it not primarily as a stressful period with the risk of burnout, but as a transformative journey fostering significant learning and growth (Atherley, 2021) and potentially serving as a critically intensive learning period (Kilminster et al., 2011). Atherley et al. (2019) emphasise the importance of viewing transitions as developmental, encouraging reflection and the development of transferable skills. Research adopting this developmental approach has shown that, for junior doctors, the transition can be experienced as both a rite of passage (Lefroy et al., 2017) and as an eye-opening period (Carlsson et al., 2023), underscoring its importance in their professional journey. From a workplace learning perspective, transitions such as these are not just about applying previously acquired knowledge but about learning through participation in practice (Billett, 2011). Workplace learning occurs as individuals engage in and make sense of their work, shaped by both the demands of the environment and their own agency in learning (Billett, 2016).

While much research has, in different ways, explored junior doctors’ experiences of the transition, less attention has been given to the perspectives of other key stakeholders. In Sweden, the medical internship (*allmäntjänstgöring*, in Swedish) serves as a bridge between undergraduate studies and licenced medical practice and is overseen by internship programme directors (PDs). PDs play a crucial role as educators and key influencers in shaping the medical education landscape, yet their perspective on the transition from student to doctor has not yet been examined in depth. Given their close interactions with interns and their influence over the internships’ educational content, PDs’ insights offer a unique vantage point for understanding how the transition to clinical practice can be seen as developmental. Such insights could lead to a more balanced understanding of the transition phase and provide a foundation for enhanced educational practices (Kumar et al., 2019).

### Aim

The aim of this study was to explore how internship PDs percieved the transition from student to doctor from a developmental perspective. The research question was therefore formulated as: How do internship PDs perceive the meaning of the medical internship?

### The medical internship and role of internship PD

In Sweden, the 5.5-year undergraduate medical programme is followed by a medical internship which extends over 18–21 months. The internship includes rotations across four areas: medicine, surgery, psychiatry, and primary care, with each rotation ranging from three to six months. The internship is considered as an introductory service to clinical work and primarily includes clinical work where interns participate in patient care as junior doctors (Socialstyrelsen, 1999). Additionally, weekly lectures and seminars provide theoretical education. Interns are assigned a personal supervisor during each rotation, and the internship often include individual as well as group mentoring sessions for reflection and discussion. Interns are assessed through mandatory ‘sit-ins’ with specialist doctors and through a final written exam covering the main rotations. The Swedish National Board of Health and Welfare legally mandates the internship’s learning objectives, while the local hospitals providing internship positions are responsible for determining the specific content and format (Socialstyrelsen, 1999). Upon completion, interns obtain full certification (licencing), qualifying them to apply for residency, i.e. speciality training. Typically, hospitals appoint one or more senior doctors as PDs to manage the internship. These directors are tasked with coordinating interns’ clinical placements and educational activities and providing support to both interns and their supervisors. Also, they guard interns’ progress and take actions if necessary. The exact responsibilities of the PDs can vary based on local practices. In smaller county hospitals, PDs may also hold the position of head of interns, responsible for decisions such as salary and management, whereas in most hospitals, these duties are typically assigned to designated head of interns (SLF, 2020).

### The Swedish healthcare context

The Swedish healthcare system is publicly funded, with universal access to care and a relatively high ratio of physicians per capita compared to many other countries (OECD, 2019). Compared to healthcare systems with greater resource constraints, Swedish interns may face lower patient loads and operate in a healthcare system that emphasises multi-professional collaboration and team-based care. However, as in many countries, tensions exist between service provision and educational priorities, particularly in balancing interns’ learning needs with the demands of efficient healthcare delivery (Carlsson et al., 2022).

## Methods

Situated in a constructivist tradition, we used individual in-depth interviews to explore the research question. Data collection and data analysis were informed by principles of the phenomenographic approach (Åkerlind, 2005; Marton & Booth, 1997; Stenfors-Hayes et al., 2013). Phenomenography was considered particularly suited in this study considering how it allowed us to explore the different ways in which PDs perceived the meaning of the medical internship, capturing the diversity of their experiences. Additionally, the phenomenographic approach offers a robust framework for linking empirical findings to educational change (Stenfors-Hayes et al., 2013).

### Phenomenography

The phenomenographic approach is used in various research fields and focuses on the *variation* of how phenomena are experienced, or rather; percieved (Marton & Booth, 1997). In phenomenography, experiences are seen as shaped both by external input from the phenomenon itself, and by the previous experience of the individual (Åkerlind, 2012). Phenomenography thus assumes a non-dualist ontology, positing that there is no separation between the individual and the world. Instead, experiences are seen as relational, formed through the interaction between individuals and their environment (Åkerlind, 2024). Whereas each person’s experience of the phenomenon is considered unique, similarities between individual experiences can be identified as they relate to the same phenomenon. Ways of experiencing, also known as *conceptions* in phenomenography, can therefore be described. These go beyond more traditional thematic descriptions in that they incorporate both the content of a perception and how the perception relates to other ways of experiencing the same phenomenon. Conceptions are then organised into analytical *categories of description* (Marton, 1981). The categories of description are not seen as capturing individual or isolated ways of experiencing, but as representing a comprehensive understanding of the phenomenon shared by all study participants (Collier-Reed et al., 2009). A set of descriptive categories collectively forms the phenomenographic *outcome space*, which includes a representation of how the categories relate to each other internally.

### Participants

Prior to data collection, an estimation of the number of participants needed to achieve sufficient information power was conducted (Malterud et al., 2016). The participants belonged to a clearly defined group with varying levels of experience. Additionally, the interviewer (YC) was an intern herself at the time of data collection, familiar to most participants through her previous research, and well acquainted with conducting semi-structured interviews, facilitating clear communication between researcher and participant. As a result, including 10–15 participants was deemed both feasible to include and sufficient to reach information power. Participants were purposefully selected based on the following criteria: (1) holding their position as a PD for at least one full year; (2) not working at the same site as the primary researcher (YC) to avoid conflicts of interest; and (3) working in regions geographically close to the research team, as face-to-face interviews were preferred. PDs who met these criteria were contacted via email and invited to participate voluntarily. From those who expressed interest, a strategic selection was made to achieve a high degree of variation, also known as maximum-variation sampling (Kuper et al., 2008). In total, twelve participants were included in the study. There was a considerable variation among the participants in terms of age (31–67 years), gender (five women, seven men), geographical region (six regions), hospital setting (two university hospitals and ten county hospitals), area of specialisation (covering both primary and secondary care), and experience in the role (ranging from one year to 16 years as a PD, with a median of four years). None of the included PDs held dual roles as both PD and head of interns.

### Data collection

In line with the phenomenographic approach, interviews were structured around several pre-determined topics outlined in an interview guide, progressing from ‘what’ questions, exploring content and practices, to ‘why’ questions, exploring awareness and understanding of learning (Åkerlind, 2005). To encourage a deep exploration of each question and to ensure a mutual understanding of the phenomenon, participants were frequently prompted with follow-up questions such as: ‘How do you mean?’; ‘Can you give a concrete example of that?’; and ‘Do I understand you right when you say…?’. Most interviews took place at the participants’ workplace, except for two conducted online due to the covid-19 pandemic. The interviews lasted between 38 and 64 min, with a median duration of 50 min. The main author (YC) conducted, audio-recorded, and transcribed all interviews verbatim.

### Data analysis

The data underwent phenomenographic analysis (Åkerlind, 2005; Marton & Booth, 1997) and was conducted in close collaboration between YC, FO, and ML. Initially, both YC and FO independently read the transcribed interviews. This was followed by a process in which text passages relevant to the research question were grouped into emanating *categories of description* (from now on referred to as categories) that reflected similar ways of understanding. At this stage, the essential meaning of each category was articulated, ensuring that each category was distinct and that an inclusive hierarchical structure was beginning to form. Parallel to the construction of categories, the logical relationship between the categories was analysed, which led to the construction of the *dimensions of variation*. These dimensions represent the critical aspects, or contrasts, discerned between categories, highlighting attributes or characteristics that vary across the data. Preliminary categories were then subject to discussions in the research team to clarify the relationships between the categories and the dimensions of variation, forming the phenomenographic *outcome space*. In the final phase, attempts were made to remove or replace each category and dimension within the outcome space, however, all were deemed essential. The analysis was considered complete once the categories met the criteria of being distinct, parsimonious, and relating clearly to the data and each other (Marton & Booth, 1997).

### Reflexivity

Reflexivity, a critical element in qualitative research, involves researchers continuously examining how their background, assumptions, and interactions influence the research process and outcomes (Olmos-Vega et al., 2023). In this study, all members of the research team—except FO—had firsthand experience of the transition from student to doctor, having previously been interns. As the first author, I was an intern at the time of data collection, which likely influenced both data collection and analysis, as certain aspects of the internship resonated more strongly with me. Additionally, during this period, the research team was finalising two studies on interns’ experiences of the transition (Carlsson et al., 2022, 2023), which further shaped our approach to data collection and interpretation in the current study. For example, our familiarity with the internship allowed us to craft contextually relevant interview questions and probe deeper into aspects we knew could be particularly meaningful or challenging. At the same time, this insider perspective required us to remain reflexive throughout the analysis, ensuring that our own experiences did not overshadow the range of perspectives expressed by the PDs. To explore whether being interviewed by an intern influenced how PDs shared their views about the internship, they were asked at the end of the interview to reflect on this experience. All participants expressed feeling comfortable and confident in sharing their honest views.

Regarding the rest of the research team, FO was a senior medical student, still anticipating the internship. ML was a resident in oncology and an educator in both undergraduate and postgraduate medical education. SB was a practicing family physician (specialist), and AN, a former head of interns, was the head of the orthopaedic department at the same hospital. Interestingly, differences in experience within the research team aligned with variations in how the transition was conceptualised. The two more junior doctors (YC, ML) emphasised the internship as a phase that should provide both structured education and integrated workplace learning, whereas the two senior doctors (SB, AN) placed greater importance on developing efficiency and contributing to patient care. These differing perspectives served as a reminder to remain open to multiple interpretations of the data.

None of us were responsible for training either interns or PDs, and we intentionally excluded PDs from our own hospital as informants to avoid prior relationships unduly influencing the analysis. As HPE researchers, we have previously drawn on theories of workplace learning and work-integrated learning, which inevitably shaped our analytical lens. Rather than striving for neutrality, we acknowledge that our perspectives shaped how we framed questions, interpreted responses, and identified patterns in the data.

### Ethical considerations

The study was performed in accordance with the ethical standards as laid down in the Helsiniki Declaration (WMA, 2013). Ethical approval was sought and obtained from the regional ethical committee in Gothenburg (Dnr 285 − 17). All participants gave informed consent to partake after receiving both verbal and written information about the purpose of the study. They were also informed that they could withdraw their participation at any time without needing to justify their decision. During transcription, interviews were anonymised, and quotes used in the manuscript contain no information that could compromise participant anonymity.

### Findings

The phenomenographic analysis gave rise to three distinct ways of perceiving the meaning of the medical internship: The internship as an education, The internship as working as a doctor, and The internship as a space for learning through work. Additionally, seven dimensions of variation were identified along each of which the internship was understood from a basic to a more nuanced view: Learning focus, Educator strategy, Internship mandate, Learning driver, Degree of patient responsibility, Role of PD, and Internship structure significance. The categories and dimensions together form an outcome space shown in Table 1, illustrating the PD’s perception of the meaning of the medical internship.

**Table 1:**
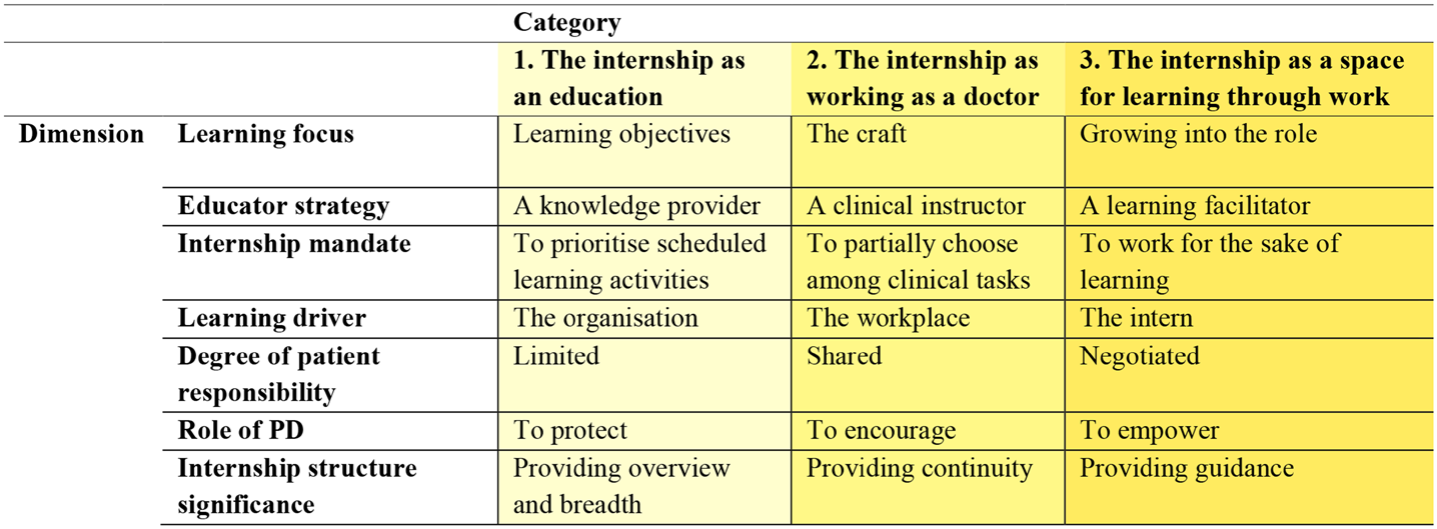
The hierarchical and inclusive outcome space illustrating programme directors’ perception of the meaning of the medical internship, where higher-order categories encompass and build upon lower order ones

### Category 1: The internship as an education

In the first category, the PDs perceived the internship as an educational phase primarily focused on *fulfilling the learning objectives* (dimension: Learning focus) necessary for all interns to become licenced doctors. The PDs acknowledged the need of various learning activities designed to enhance interns’ medical knowledge for them to meet the government-mandated standards. To keep track of intern’s progress, some PDs described how they had developed tailored tools that aligned with these objectives.

*We have created a booklet with the different learning objectives so that we make sure that we don’t miss anything. I go through the booklet with the intern in the middle of the internship*,* to kind of figure out “so what boxes do we have left to tick here?”* (P2).

Learning was seen as happening through structured activities such as lectures and seminars, where the educator - whether inside or outside the clinic - was seen as *a knowledge provider* (dimension: Educator strategy), ‘transferring’ medical knowledge to the interns, not always in direct relation to interns’ daily work.

*The internship should also allow for things that might not necessarily benefit the clinic*,* like educational elements - that they have lectures*,* and that there is education in other ways as well.* (P4)

According to the PDs, the internship gave the interns authority to *prioritise scheduled learning activities* (dimension: Internship mandate) over their clinical duties when necessary, ensuring their educational needs were protected and not overshadowed by clinical demands. This was often contrasted with other junior doctors not yet enrolled in the internship programme, who were not afforded the same mandate.

*[The interns] have their programme director*,* the entire internship structure*,* they work according to well-defined learning objectives*,* they have their supervisor*,* erm … This gives them like a mandate to leave [the clinic] to attend lectures and seminars*,* whereas the other junior doctors are completely at the mercy of the clinic where they are hired.* (P3)

The *internship organisation* was itself described as an important catalyst for learning (dimension: Learning driver), both by ensuring that the learning objectives were actually met and by its mandatory and structured checkpoints, such as ‘sit-ins’ and the final internship exam. As one participant noted, these checkpoints were particularly effective, as *‘when preparing for the final written internship exam*,* [the interns] actually learn a great deal’* (P1). In this category, PDs’ percieved interns primarily as learners, emphasising their status as unlicensed practitioners. Consequently, interns were not expected to make independent decisions or assume substantial patient responsibility, hence *limited* (dimension: Degree of patient responsibility). This stance was articulated by one participant, who pointed out that *‘formally*,* they don’t bear any responsibility*,* it’s almost as if they’re not allowed to make their “own” decisions’* (P6). In the same vein, the PDs understood their role as a *protection* against a too high clinical workload and the sometimes harsh realities of healthcare (dimension: Role of PD).

*What I also think is important is that it’s clear who they can turn to and that someone listens*,* during the internship. That there are several kinds of ‘safety-nets’*,* a programme director and a supportive organisation around them.* (P5)

The PDs further underscored the importance of experiential learning, noting that interns should be exposed to a variety of patients across different clinical settings, engaging in many different tasks, to learn as much as possible. In other words, the internship should provide an *overview and breadth* of medical knowledge (dimension: Internship structure significance). As one PD remarked, *‘having seen different parts of a very complex healthcare system is wise*,* regardless of where you end up later’* (P8) and thereby highlighting the belief in the internship as a preparatory experience.

In summary, the internship was here seen as an educational phase where interns, primarily viewed as learners, engaged in structured learning activities and assessments, preparing them for their licenced medical practice.

### Category 2: The internship as working as a doctor

In the second category, the internship was perceived as a phase of ‘practical doctoring’, where interns learned *the craft* of a doctor by putting theory into practice (dimension: Learning focus). The main purpose of the internship, according to the PDs, was to *‘produce well-functioning clinicians’* and ensure that interns became *‘employable’*. While recognising the value of theoretical education, the PDs argued that the most important learning happened during the interns’ daily activities, offering direct and hands-on experiences in the clinical setting.

*So*,* for me it’s mostly a skill training*,* like schooling them in how to be a doctor*,* where they should learn both what it’s like to be a colleague and what it’s like to be a doctor.* (P2)

In this category, the interns were described as future colleagues, who learnt to develop decision-making skills and take on increasing responsibility. *The workplace* was seen as the primary driver of this learning process, encompassing the interns’ different tasks, colleagues, superiors, and patients (dimension: Learning driver). The PDs underscored that *‘the job is the education’* and the necessity for interns to engage directly with the patients for the internship to serve its purpose.

*The only way to learn what you need to know as an intern is to produce healthcare. So*,* you have to meet patients and deal with patients to learn what you need to know.* (P6)

Although patient-related tasks were at the core of this category, the PDs believed it was important for interns to have some autonomy in *choosing among clinical tasks* (dimension: Internship mandate). This flexibility allowed interns to address percieved knowledge gaps while also letting their interest and motivation guide them. For instance, one PD shared an example of how an intern’s initiative led to significant hands-on experience:

*I had an intern who*,* during her surgical rotation*,* told everyone*,* “I want to do minor surgeries”*,* which meant that everyone called her. So*,* she ended up performing a huge number of surgeries.* (P4)

The PDs also pointed out that while interns were expected to take on and accomplish an increasing number of tasks, and increasingly difficult such, their practice was overseen by more experienced doctors. The training thus involved a certain degree of patient responsibility, yet it was not absolute; or as one PD said: *‘the supervisor is also responsible*,* so that the interns can practice in safety’* (P2), capturing a *shared responsibility* (dimension: Degree of patient responsibility). Furthermore, the PDs emphasised that, due to interns’ lower position in an implicit hierarchy, interns should continually seek support and ask for help. This junior status thus came with the need of always having a more experienced colleague around to instruct and show *‘how to do things’*, where the educator was viewed as *a clinical instructor* (dimension: Educator strategy).

*The intern always works with a more experienced colleague*,* often a specialist or a senior physician*,* and […] that’s where it becomes very clear that it is an apprenticeship profession – ‘do as your master’.* (P11)

The PDs saw their role as motivators and believed it was their responsibility to *encourage* the interns to dare face challenges and thus build their confidence (dimension: Role of PD). This involved a balance between support and a nudge towards increased autonomy, essential to help interns transition from students to practitioners.

*It’s all about constantly encouraging them and giving a sort of gentle push in the back so that they dare taking that emergency call. It’s all about pushing them.* (P3)

Regarding the structure of the internship, the PDs particularly emphasised the importance of longer rotations *providing continuity*, allowing interns to become legitimate members of the workplace by building meaningful relationships with staff, patients, and supervisors (dimension: Internship structure significance). They argued that too short rotations might prevent interns from properly settling in and assuming significant roles.

*So*,* the learning curve is extremely steep when you stay at one workplace a bit longer – much steeper than if you constantly move from one place to the other.* (P9)

In summary, the PDs here percieved that the internship effectively prepared interns to work as doctors by instructional hands-on learning in the healthcare team over a longer period of time.

### Category 3: The internship as a space for learning through work

In the third and final category, the internship was seen not merely as a preparatory training or a job experience, but as a period of work-integrated learning in an environment that fostered the best possible learning in a clinical setting. The learning focus thus extended beyond simply meeting learning objectives or learning the craft; it emphasised a more comprehensive development as doctor, both personally and professionally – to *grow into the role* (dimension: Learning focus).

*You don’t just go to work and then suddenly you’re a doctor*,* and then you go home and go back to being yourself. The medical profession largely involves using your own persona as a tool. And [the internship] is about finding some kind of harmony in the ‘self’ as a doctor.* (P3)

The *intern’s* own motivation to learn was considered a key element (dimension: Learning driver). Despite structured opportunities and resources provided by the internship itself or by the workplace, the ultimate learning still depended on the intern’s engagement in the daily work, and the PDs saw it as the intern’s responsibility to harness the learning possibilities afforded by the internship.

*The [intern’s] inner will: “Now I really want to work as a doctor*,* I’ve spent five and a half years at the university. Now I want to face reality*,* I want to meet patients*,* I want to take care of them myself”. This commitment or fundamental interest in getting to work with what they have trained for so long*,* I believe that this inner drive is the most important thing.* (P12)

A key aspect of the internship, according to the PDs, was to allow interns to *work primarily for the sake of learning* (dimension: Internship mandate) by ensuring that interns’ placements and tasks were guided by educational, not service, needs. As one PD put it, *‘clinical work should be synonymous with educational work’* (P6), underscoring the ideal that every clinical encounter should be a learning opportunity. If this ideal wasn’t upheld, the PDs recognised the risk that interns, with high service demand, could be relegated to performing administrative tasks that *‘anyone could really do’* (P3). To achieve a learning-driven practice, the PDs emphasised the importance of curating tasks conducive to learning and providing interns with constructive, high-quality feedback—not just as one-way instruction, but by creating a space for dialogue and reflection. In line with this, the PDs saw the interns’ various teachers and educators not just as knowledge providers or instructors, but as *facilitators of learning* in a broader sense (dimension: Educator strategy). This approach shifted the focus from merely imparting procedural knowledge to fostering a supportive dialogue that promoted holistic development.

*As we see it*,* these conversations are not about learning which blood tests to take for a certain diagnosis*,* or how to perform an appendectomy. Those are more instructions. Instead*,* we view mentorship as sitting down and asking*,* “How are you doing? Are you getting enough support? Do you feel that you’re making progress?”* (P10).

Furthermore, the PDs acknowledged the importance of gradually entrusting interns with increasing responsibility for them to grow as doctors. This increasing responsibility was based both on the intern’s demonstrated competence and ability to judge when to seek help and when to manage independently; hence *negotiated* (dimension: Degree of patient responsibility).

*Interns who feel safe and secure can be the only ones who actually see the patient on site*,* make a decision on site*,* and then the patient goes home*,* without having met another doctor. But the intern can always consult their supervisor afterwards.* (P6)

Importantly, one PD pointed out that *‘it is not as if something magically happens the day that interns become licensed doctors’*, but that *‘it is a process’* (P6), illustrating the need of allowing interns to be increasingly accountable for patient care during the internship. The PDs considered it their responsibility to *empower* the interns (dimension: Role of PD), by encouraging them to independently handle challenges while knowing that support was readily available.

*They know that I respond to emails right away and answer the phone […] and that I’m available. Just knowing that can sometimes be a reassurance*,* and they might feel more confident to first discuss matters with their superior*,* and then their line-manager […] and if things still don’t work out*,* I’ll step in.* (P2)

The PDs thought of the internship as *providing guidance* to the interns (dimension: Internship structure significance), in helping them navigate the complexities of the medical profession. They highlighted the need of both breadth and continuity, alongside in-depth dialogue with supervisors and mentors focused on the intern’s progress. They emphasised that the internship’s ideal aim was to shape well-rounded physicians not only skilled in clinical procedures but also reflective and adaptive in their practice.

*To kind of pilot the interns to understand what it means to be a doctor*,* how to function as a doctor*,* what the challenges of the medical profession are*,* how one can improve*,* how one can delve deeper into certain knowledge areas and what one’s weaknesses are.* (P9)

In summary, the internship was in this category envisioned as a dedicated space for work-integrated learning, where interns could profoundly develop not only their medical knowledge and practical skills, but also their personal and professional *self* as doctors.

### Relationship between categories

**Fig. 1:**
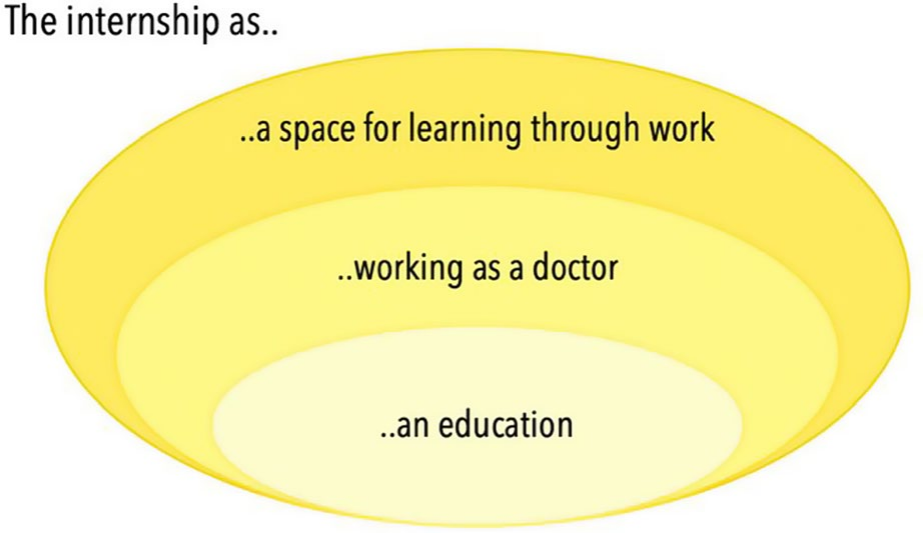
The outcome space visualised inclusivly and hierarchically

The three categories forming the outcome space are shown in Fig. 1 with their hierarchical relationship illustrating increasing complexity, where the first category, Internship as an education, represents a more basic conception, while the third and final category, Internship as a space for learning through work, reflects a more nuanced and integrative understanding. Although this hierarchy indicates an increasing complexity, it does not imply a value-judgement, i.e. that lower-order categories are incorrect or less valuable (Åkerlind, 2024). Rather, it illustrates the range of perceptions among participants, from foundational to more advanced, with each category building upon the previous one. The categories thus span a continuum, from seeing the internship as a phase of acquiring medical knowledge before becoming a licenced doctor (category 1), to learning the craft through actively participating in healthcare delivery (category 2), and finally, to seeing it as structured opportunity for comprehensive learning through work, where patient care responsibility is balanced with personal and professional growth (category 3). Each category should not be viewed in isolation but as part of an inclusive hierarchy, where lower-order categories are encompassed within the higher-order ones. If the first category highlights the intern as primarily a learner, and the second as a functioning clinician, the third category presents the intern’s dual role as learner and clinician, *a learning clinician*.

## Discussion

This study aimed to explore the transition from student to doctor from a developmental perspective, by using a phenomenographic approach and the Swedish medical internship as a proxy for the transition. Our findings show that the PDs perceived the meaning of the internship in three distinct ways: as an education; as working as a doctor; and as a space for learning through work. The perspective of a learning clinician, as demonstrated in category 3, offers a holistic understanding of the internship, marked by the interplay between clinical work and its inherent learning opportunities, in that learning ideally happens during work and through work, when the work environment is designed to be conducive to learning.

This study offers a novel perspective by exploring PDs’ views on the transition from student to doctor, complementing previous research on junior doctors’ experiences. While junior doctors often describe this phase as both demanding and formative (Brennan et al., 2010; Carlsson et al., 2023) highlighting emotional strain and uncertainty (Prentice et al., 2020), PDs recognised it as a key period of professional development. They viewed challenges—such as hands-on work and increasing patient responsibility—as essential for growth, provided they were managed within a supportive learning environment. Previous studies suggest that junior doctors sometimes feel overwhelmed by service demands and struggle to protect their learning opportunities (Atherley et al., 2019). However, PDs in this study were strong advocates for interns’ right to learn, which was notable given their seniority and the competing pressures of service delivery. Rather than seeing service and learning as conflicting priorities, PDs emphasised that meaningful learning should happen through clinical work itself—especially when daily tasks are intentionally structured to support education. By considering both perspectives—those of junior doctors navigating the transition and those of PDs shaping the learning environment—this study presents the transition as a shared developmental journey: one marked by challenges but ultimately about growth. This dual perspective underscores the importance of balancing a protected learning space with active participation in clinical practice, finding the sweet spot balance between challenge and support (O’Brien, 2018).

One particularly interesting finding in our study was the concept of the internship as a *mandate* for learning, where interns were allowed to be driven by their need to learn rather than by the demands of efficient patient care. This mandate seemed to enable interns to focus on their development—whether by attending learning sessions, selecting tasks that fostered learning, or stepping away from routine clinical duties with little educational value. Without maintaining this learning mandate, interns might be at risk of becoming overwhelmed by service tasks and increased patient responsibility. The mandate appeared important for creating a space where interns could thrive both as clinicians and learners. This notion aligns with van der Zwet et al.’s (2011) discussion of clinical work as synonymous with learning opportunities. Moreover, our most advanced category, *a space for learning through work*, closely mirrored van der Zwet et al.’s concept of a *developmental space* which emphasised the socio-cultural context to truly *‘learn from doing’* (p. 372). Both studies identified similar key elements for fostering this space: longer placements allow learners to build relationships and settle into their roles, recognition as legitimate team members, progressively independent practice, and readily available supervisors offering individualised one-to-one supervision.

Notably, the PDs’ emphasis on protecting interns’ right to learn may also be shaped by the broader healthcare and educational context in Sweden. One possible influencing factor is that interns are centrally funded at each hospital rather than being financially dependent on the clinics where they rotate. While clinics oversee interns’ supervision and integration, they do not bear the cost of their presence, reinforcing the idea that interns are first and foremost learners, rather than essential service providers. Additionally, Sweden’s relatively flat organisational culture, combined with its state-funded healthcare system, may foster a workplace where learning is prioritised and openly supported by senior staff—contrasting with more hierarchical systems, where junior doctors may struggle to assert their learning needs.

Nonetheless, elements in our findings resonate with Billett’s theoretical insights into workplace learning. Billett argues that learning opportunities are maximised not merely through exposure to work, but through an intricate balance of guidance, support, and real responsibilities (Billett, 2016). In our findings, this is evident as the PDs perceive the internship not just as a time for more education (category 1) or learning the craft (category 2), but as a complex yet structured space where learning is interwoven with daily clinical tasks (category 3). The PDs’ acknowledgement of the internship as a space for learning through work suggests a developmental trajectory where interns are expected to evolve from novice learners to competent and safe practitioners who are reflective and proactive. It is arguable that such development is crucial not only for clinical competence but for fostering a sense of agency and self-efficacy among junior doctors.

To enhance the developmental potential of the transition to clinical work, faculty developers and other stakeholders might consider implementing regular and structured opportunities for reflection that can help new doctors to assimilate their experiences and insights, fostering a deeper learning (Park et al., 2024). Additionally, supportive mentorship initiatives could provide junior doctors with the guidance needed to navigate the complexities of the transition from student to doctor (Winkel et al., 2023). Lastly, regular and constructive feedback allows the learner to understand their developmental progress and identify areas for improvements, aligning with Billett’s emphasis on active engagement in the learning process (2016). The evolving nature of healthcare and medical education implies a need for ongoing research regarding the transition to clinical practice. Future research should explore this transition from the perspectives of other stakeholders, such as senior medical students, supervisors, and even patients, to provide an even more comprehensive understanding of this phase.

### Methodological considerations

Like all studies, this one has limitations and could have been approached differently. Importantly, we do not claim that this study offers a complete picture of the transition from student to doctor. A phenomenographic outcome space will always be partial, reflecting only a portion of the many ways in which a phenomenon may be experienced. However, the goal of a phenomenographic study is to develop an outcome space as complete as possible (Kullberg & Ingerman, 2022). One way to achieve this is through collaborative analysis, allowing the research team to better capture all relevant nuances in the data (Åkerlind, 2005), which we have done in this study. In line with the phenomenographic approach, the interviews were viewed as forming a *pool of meanings* at a collective level. This means that individual participants may not necessarily recognise their own contributions within the outcome space (Collier-Reed et al., 2009). Consequently, using member checking to ensure trustworthiness is not appropriate for this approach and was therefore not conducted. However, the findings have been shared with relevant stakeholders, including heads of interns and internship PDs, to spark discussions about the transition to clinical work. The findings resonated strongly within the group, particularly the final category – a space for learning through work – which was highlighted as an ideal design for the internship. This resonance reflects what Stalmeijer et al. (2024) describe as transferability through resonance, where qualitative findings evoke a sense of familiarity or recognition among those embedded in the studied context, suggesting that the findings authentically represent their lived reality. Additionally, we have sought to provide thick contextual detail to allow readers to consider the relevance of our findings to their own settings.

## Conclusion

In conclusion, can we fully prepare students for clinical practice? It seems difficult to do so, as real-world clinical scenarios are unpredictable and require learning through hands-on experience. While the challenges of beginning clinical work are most likely inevitable, this study suggests focusing on fostering an environment that promotes active engagement, reflective practice, and robust support systems. Such an approach may help alleviate some of the difficulties of this phase, helping to make it more meaningful. Additionally, granting junior doctors the mandate to learn through work could empower them as proactive learners and enable them to become capable, confident, and reflective practitioners.

## Data Availability

No datasets were generated or analysed during the current study.
